# Exclusive Biosynthesis of Pullulan Using Taguchi’s Approach and Decision Tree Learning Algorithm by a Novel Endophytic *Aureobasidium pullulans* Strain

**DOI:** 10.3390/polym15061419

**Published:** 2023-03-13

**Authors:** WesamEldin I. A. Saber, Abdulaziz A. Al-Askar, Khalid M. Ghoneem

**Affiliations:** 1 Microbial Activity Unit, Microbiology Department, Soils, Water and Environment Research Institute, Agricultural Research Center, Giza 12619, Egypt; 2 Botany and Microbiology Department, Faculty of Science, King Saud University, Riyadh 11451, Saudi Arabia; 3 Seed Pathology Research Department, Plant Pathology Research Institute, Agricultural Research Center, Giza 12619, Egypt

**Keywords:** biopolymer, fungal polymer, exopolysaccharide, optimization, fermentation, modeling, signal to noise ratio

## Abstract

Pullulan is a biodegradable, renewable, and environmentally friendly hydrogel biopolymer, with potential uses in food, medicine, and cosmetics. New endophytic *Aureobasidium pullulans* (accession number; OP924554) was used for the biosynthesis of pullulan. Innovatively, the fermentation process was optimized using both Taguchi’s approach and the decision tree learning algorithm for the determination of important variables for pullulan biosynthesis. The relative importance of the seven tested variables that were obtained by Taguchi and the decision tree model was accurate and followed each other’s, confirming the accuracy of the experimental design. The decision tree model was more economical by reducing the quantity of medium sucrose content by 33% without a negative reduction in the biosynthesis of pullulan. The optimum nutritional conditions (g/L) were sucrose (60 or 40), K_2_HPO_4_ (6.0), NaCl (1.5), MgSO_4_ (0.3), and yeast extract (1.0) at pH 5.5, and short incubation time (48 h), yielding 7.23% pullulan. The spectroscopic characterization (FT-IR and ^1^H-NMR spectroscopy) confirmed the structure of the obtained pullulan. This is the first report on using Taguchi and the decision tree for pullulan production by a new endophyte. Further research is encouraged for additional studies on using artificial intelligence to maximize fermentation conditions.

## 1. Introduction

The ubiquitous yeast-like fungus, *Aureobasidium pullulans* (de Bary) G. Arnaud (1918), is found in various conditions, especially in the phyllosphere, as an endophyte or epiphyte of a broad extent of plants, without producing disease symptoms. Biotechnologically, *A. pullulans* is regarded as a highly important fungus in the production of various biomolecules, e.g., siderophore, and enzymes [1,2,3,4]. The fungus is polymorphic and generally regarded as non-toxigenic and non-pathogenic [5]. The biosynthesis of pullulan (poly-α-1,6-maltotriose) by *A. pullulans* is the main spot [6,7].

The genus *Aureobasidium* is an ascomycetous yeast-like fungus that comprises 27 taxa (14 species and two varieties). Among these species, *A. pullulans* (de Bary) G. Arnaud is the most studied [6]. *A. pullulans* is a widely distributed black, yeast-like fungus that can naturally exist in various environments, including the phyllosphere [1,3]. It can be found as an epiphyte and/or endophyte of a vast array of plants (e.g., apple, cucumber, green beans, grape, stored barley grain cabbage, and coconut water) without developing diseases [1,2,4].

Pullulan is a biodegradable polymer derived from natural resources and can be considered a renewable resource and environmentally friendly material; it has potential uses in various industries, food packaging, medical devices, and cosmetics [6]. Pullulan is an unbranched extracellular polymer, which is neutral (like dextran, cellulose), water-soluble, and viscous polysaccharide. The alpha-glucan consists of units of glucose with arranged in a linear structure, i.e., three units of glucose molecules are linked by α-1,4 glycosidic bond to form maltotriose units, which are repeatedly polymerized by α-1,6 glycosidic bond on the terminal glucose, thus pullulan known as α-1,4-; α-1,6-glucan [8,9].

Pullulan helps cells withstand dehydration and predator, and also allows for an easier exchange of substances in and out of the cell [10]. Low concentrations of pullulan form high-viscosity solutions and can be made into fibers and films, making it ideal for coating food and pharmaceuticals. It is available commercially as a tasteless polymer, used in food and non-food industries as an additive, processing aid, thickener, stabilizer, emulsifier, and gelling agent. Additionally, pullulan is used in various applications, including edible films for breath fresheners, pharmaceuticals like retard tablets, capsules, and microcapsules, and industries such as cosmetics, textiles, and paper [6,7,8,10].

Several attempts are being done to manipulate pullulan biosynthesis through genetic engineering. For instance, overexpression of the gene encoding for the pullulan synthase enzyme has been reported to increase pullulan production [11]. However, genetic engineering methods can be time-consuming and expensive, and the stability of the modified strains can be a limitation. Optimization of the medium composition, as well as the different process parameters (e.g., temperature, pH, and agitation) for pullulan production by varying the level of each variable, can be challenging and have conflicting effects; this is due to the biosynthesis process needs an accurate balance of the fermentation conditions and very sensitive to the interactions among components [12,13,14].

Because of the drawbacks of genetic engineering, and alteration of the medium components (that may lead to an inconstant supply of pullulan due to uncontrolled factors), Taguchi’s experimental design can deal with such issues. Taguchi’s method is a robust statistical design that optimizes process performance and quality using fractional factorial design and multi-parameter optimization. It is a straightforward and organized approach for finding the optimal level of each factor while maximizing the response with a limited number of experimental runs on multiple variables [15,16]. The Taguchi method originated from expanded factorial and Latin squares (L) designs, known as orthogonal arrays, which are matrices where every pair of columns contains the same number of each possible pair of factors [16,17].

The goal of orthogonal arrays is to optimize the signal-to-noise (*S/N*) ratio of responses rather than the responses themselves, reducing process variability [18]. The *S/N* ratio calculates the response’s deviation from the target. It represents the mean value as signal and the standard deviation as noise. The objective is to minimize variability by amplifying the *S/N* ratio. This is what differentiates the Taguchi method from traditional statistical techniques. Taguchi categorizes the *S/N* ratio into three types, i.e., nominal is better, larger is better, and smaller is better, depending on the desired response [15,17]. The *S/N* ratio replaces the response in determining the optimal control factor levels and disregards variations caused by uncontrollable conditions [15]. To our knowledge, none of the previous attempts at pullulan biosynthesis used Taguchi’s approach.

A decision tree (DT) is a supervised machine learning and data mining algorithm represented as a tree-like structure. Each node represents a feature, branches represent decision rules, and leaves represent outcomes. The aim is to predict the target variable by learning decision rules from data features. The tree is constructed by starting at the root node and then recursively partitioning the data into subsets based on the values of the input features. The result is a tree where each leaf node represents a class label, and each internal node represents a test of one of the input features. Decision trees can also be used for other tasks, such as regression and clustering. They are commonly used in decision support systems and operations research. It is essential to have proper data collection and annotation to use a decision tree. The DT can also be used to predict the optimal process parameters for new conditions, such as changes in the composition of the inoculum or changes in the configuration of the substrate [19,20]. It is worth noting that the specific details of the implementation can vary depending on the specific application and dataset. Also, the optimization should be done with caution and validation, as the decision tree can be affected by overfitting and other issues. No previous attempts applied the DT approach in pullulan biosynthesis.

Herein, we report a novel incorporation of both Taguchi’s approach and the machine learning algorithm of DT to optimize the fermentation process parameters for maximization of pullulan biosynthesis using the new endophytic candidate, *A. pullulans*. Furthermore, *A. pullulans* were molecularly identified, and the spectroscopic characterization of the resulting pullulan was investigated.

## 2. Materials and Methods

### 2.1. Isolation of Endophytic Yeast-like Fungus

The endophytic yeast-like fungus *A. pullulans* were isolated from common bean seeds. Ten healthy seed samples were collected from various Saudi Arabia provinces (Riyadh, Al-Qaseem, Al-Ahsaa, and Ha’il), lie within 23°27′ N and 27°52′ N (latitudes), and 41°72′ E and 49°58′ E (longitudes). The collected common bean pods were stored at 4 °C after labeling. Isolation of the endophytic seed-borne yeast-like fungus from seed samples was carried out following the technique proposed by Rashad et al. [21]. First, the surface was sterilized with 70% ethanol for 1 min, then 2% sodium hypochlorite for 90 s, then with 100% ethanol for 30 s, and finally, rinsed with distilled water for 10 min before drying under laminar airflow, utilizing sterilized filter paper. Potato sucrose agar (PSA) medium was used to plate 10 seeds and incubated at 25 ± 2 °C in the dark. After 4 days, colonies around each seed were picked and purified by respreading on PSA plates. The single pure colonies were obtained, which were subcultured regularly on PSA slants.

### 2.2. Identification

#### 2.2.1. Morphological Characteristics

For the morphological identification of *A. pullulans* colonies, daily observation of colony characteristics was done. Furthermore, a slide culture was made from the grown colony on PSA, which was smeared with methylene blue and observed by wet mounting using bright field microscopy. Identification of the *Aureobasidium* strain was done following the previously reported features [22,23,24].

#### 2.2.2. Molecular Identification

*A. pullulans* was identified through molecular methods. DNA was separated and amplified using polymerase chain reaction (PCR) with DNeasy Tissue Kits (from QIAGEN, Hilden, Germany). DNA concentration was determined by comparing it with a lambda DNA standard on a 1% agarose gel. The product was diluted to 20 ng DNA/µL and stored at −20 °C.

The ITS region was amplified and sequenced. The PCR reaction mixture contained 1x buffer (Promega, Madison, WI, USA), 1.5 mM MgCl_2_, 1 U Taq DNA polymerase (GoTaq, Promega), 0.2 mM dNTPs, 30 picomoles of each primer of ITS5-F (5′-GGAAGTAAAAGTCGTAACAAGG-3′) and ITS4-R (5′-TCCTCCGCTTATTGATATGC-3′), 30 ng of genomic DNA and ultra-pure water. The total volume was made up to 50 µL. The thermal cycling was performed using the Perkin-Elmer/GeneAmp^®^ PCR System 9700 (PE Applied Biosystems, Waltham, MA, USA) with 40 cycles of 30 s each (after an initial denaturation cycle of 5 min at 94 °C), annealing (45 °C for 30 s), and elongation (72 °C for 30 s), with a final extension of 7 min at 72 °C. The PCR products were purified using the Montage PCR Clean-up kit (Millipore, Burlington, MA, USA) to eliminate unincorporated primers and dNTPs. Nucleotide sequence analysis was performed using NCBI BLASTn online tool (http://ncbi.nlm.nih.gov/BLAST/, accessed on 1 January 2023) and aligned utilizing Align Sequences Nucleotide BLAST with the nr/nt database.

The taxa’s evolutionary relationships were compared utilizing the Neighbor-Joining procedure [25]. The percentage of replicate trees (1000 replicates) was compared [26]. The pairwise evolutionary distance was calculated using pairwise deletion. The ambiguous sites were excluded, and the evolutionary analyses were carried out [27].

### 2.3. Fermentation Medium and Cultural Conditions

The synthesis of pullulan was performed using the medium of Singh et al. [28] with modifications, which contained (g/L) sucrose (50), K_2_HPO_4_ (5.0), NaCl (1.0), MgSO_4_·7H_2_O (0.2), yeast extract (2.0), and pH 6.5. This medium was used for maintenance, inoculum preparation, and production of exopolysaccharide.

The inoculum of *A. pullulans* was prepared by scraping culture slant into a 250 mL Erlenmeyer flask containing 50 mL of the sterilized medium, then incubated at 30 °C, 200 rpm. After three days, this inoculum was utilized to inoculate 100 mL of the biosynthesis medium in a 500 mL Erlenmeyer flask at the rate of 10% (*v*/*v*).

Unless otherwise stated, the production conditions of pullulan were performed by incubating the Erlenmeyer flasks at 30 °C on a rotary shaker at 200 rpm for up to 7 days. After the fermentation time, the cells were removed by centrifugation at 2500× *g* for 15 min.

### 2.4. Determination of Pullulan

Pullulan content was separated and determined following the method of Singh et al. [28]. Briefly, the cells were separated from the culture by centrifugation (1400× *g* for 15 min). The pullulan in the supernatant was separated by adding two volumes of ice-chilled isopropyl alcohol whilst stirring. The mix was kept overnight at 4 °C and then centrifuged (2500× *g* for 15 min) to separate the polysaccharide, which was redissolved in deionized water, reprecipitated again with isopropyl alcohol, and washed with acetone, then deionized water, this process repeated thrice to ensure the purity of pullulan. The obtained pullulan was dried (80 °C to constant weight). The pullulan content (%) was expressed as the weight of dry pullulan produced in deciliter fermented broth.

### 2.5. Taguchi Experimental Design

Experiments of pullulan biosynthesis using *A. pullulans* were performed in 100 mL batch fermentation conditions. For optimization of the biosynthesis process, seven independent factors were assessed, representing the medium variables reported above (sucrose, K_2_HPO_4_, NaCl, MgSO_4,_ yeast extract, pH, and incubation period). The Taguchi orthogonal array was employed to find the ideal conditions for pullulan biosynthesis. The design was structured with the minimum number of runs, i.e., 3^7. Thus 27 runs were generated for the design array by utilizing seven factors with three (high, middle, low) levels (L_27_). A generic signal-to-noise (*S/N*) ratio was employed to measure experimental variation, aiming to enhance pullulan biosynthesis quality through decreased mean squared deviation. The type of function applied (larger is better) that maximize pullulan biosynthesis the next Equation (1) was used:

(1) $$S/N\;ratio=-10\times log(\Sigma (1/Y^{2})/n)$$

where *S/N* is the signal-to-noise, n is the number of observations, and *Y* is the observed data.

Taguchi’s design was experimentally performed and repeated in three different runs. ANOVA was estimated to identify the significant parameters. Both the *S/N* ratio and ANOVA analysis allow the forecast of the most favorable process parameter combinations. A confirmation experiment was carried out to confirm the obtained optimal conditions based on the parameter design.

### 2.6. Decision Tree Learning Algorithm

A parametric learning classification and regression tree was devoted to exploring the possible solutions for improving pullulan biosynthesis using the set of tested variables. The seven fermentation parameters were the input continuous predictor variables that were used to alleviate the noise resulting from the experimental procedures. The biosynthesis of pullulan was the target variable (response).

The decision tree learning algorithm with pullulan biosynthesis was created by a group of rules based on the continuous predictor in the data set using least squared errors as a node-splitting method (i.e., choosing the variable for the smallest mean squared error (MSE) and root mean squared error (RMSE) upon regression), and maximum R^2^ for selecting the optimal tree. To evaluate the prediction precision and avoid over-fitting, validation with a test set was performed at a random fraction of 0.3, where 70% of data was applied for training and the other 30% was used for testing (70/30% training/test sets) a total of 81 runs, representing three replications of the Taguchi experimental design with 27 each. The nodes continued to split until the terminal nodes could not be divided further. Deeper trees tend to have more accurate predictions, so the deepness of the DT was fixed when further changes did not decrease the mean squared error.

### 2.7. Validation of Taguchi and DT Models

The designed model was validated experimentally using three repetitions of different combinations of the seven factors created by the two models. The observed and predicted values were compared to assess the model’s accuracy in maximizing pullulan biosynthesis.

### 2.8. Spectroscopic Characterization

The isolated pullulan samples were scanned by Fourier-transform infrared spectroscopy (FT-IR) analysis with the potassium bromide pellet method [29,30]. Infrared spectra were documented on a Bruker FT-IR spectrometer (Bruker Corporation, Vienna, Austria), OPUS version 7.5, from 4000 to 350 cm^−1^ of scale with sensitivity > 20.0%, and the data were recorded as wavenumbers (*ν*, cm^−1^).

The sample was prepared for the proton nuclear magnetic resonance (^1^H-NMR) analysis by dissolving 15 mg of the obtained pullulan in 0.5 mL DMSO-*d*_6_, and drops of D_2_O solvent were added, in which tetramethylsilane (TMS) was applied as an internal standard [24]. The ^1^H-NMR spectrum was run on Bruker AVANCE III 400 MHz NMR Spectrometer (Bruker Corporation, Vienna, Austria). The chemical shifts *δ* were recorded in ppm downfield from TMS, utilizing the tolerating deuterated signals of the used solvents as an internal reference (DMSO-*d*_6_/D_2_O: *δH* = 2.516, and 4.184 ppm). ^1^H-NMR (DMSO-*d*_6_/D_2_O) (*δ*, ppm): 3.135 (s, 1H, CH), 3.302–3.323 (d, 1H, CH), 3.390 (d, 2H, CH_2_), 3.553–3.701 (d, 1H, CH), 3.767–3.797 (d.d., 2H, CH_2_), 4.694 (s, 1H, CH), 5.027–5.061 (d, 1H, CH).

### 2.9. Software and Statistical protocol

The data of the pullulan biosynthesis by *A. pullulans* are the mean of three trials with standard deviation (±SD) values. Taguchi orthogonal array, the supervised learning algorithm of the decision tree, and the mathematical analysis were performed using Minitab software (version 21, Minitab Inc., State College, PA, USA).

## 3. Results and Discussion

### 3.1. Isolation and Morphological Identification of A. pullulans

**Figure 1:**
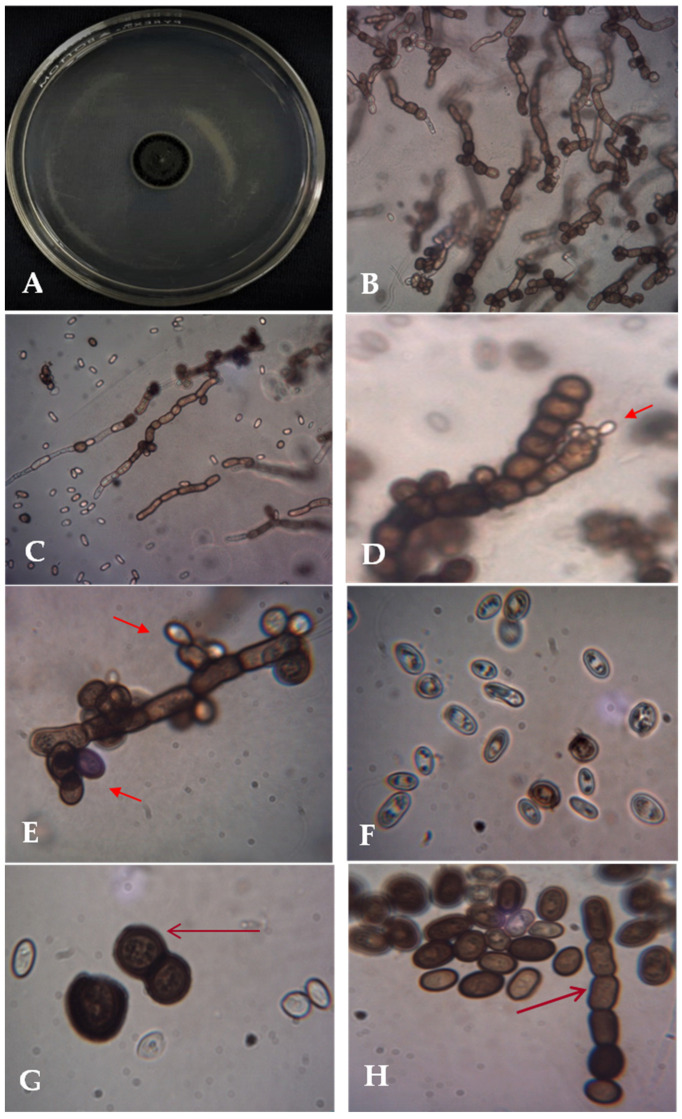
Macro- and microphotographs of *A. pullulans* AKW: the colony on PSA after 7 days at 30 °C (**A**), microscopic images of melanized hyphae with melanized conidia at 400× (**B**,**C**), conidiogenesis at 1000× ((**D**,**E**), arrowed), hyaline and melanized blastoconidia (**F**), and dark brown chlamydoconidia and chains of arthroconidia at 1000× ((**G**,**H**), arrowed).

The current trial revealed the isolation of *A. pullulans* AKW from common bean seeds as an endophytic fungus and morphologically identified. On PSA, the fungus optimally grows at 30 °C, within a temperature range of 10–35 °C. The isolated fungus produced smooth, faintly pink, yeast-like colonies enclosed with a slimy mass of spores. As time passed, the colonies turned black because of chlamydospore production. The colonies were flat, smooth, moist, yeast-like, slimy, shiny, and leathery in appearance. The surface is cream at the beginning and becomes black with time, with a grayish fringe by aging. Examination of the cell morphology of the isolate (Figure 1) by bright field light microscopy showed two types of conidia are produced. The primary conidia are smooth, hyaline, ellipsoidal, one-celled, and variable in shape and size, whereas the secondary conidia are smaller. Conidiophores can be indistinguishable, occur in between cells, or arise from short lateral branches. Endoconidia is generated within an intercalary cell and moves to a neighboring empty cell. Hyphae are clear (hyaline), smooth, thin-walled, and have transverse septa. The microscopic examination also showed the typical *A. pullulans* polymorphology with hyphae, blastospores, and chlamydospores. Both colony features and microscopic morphology were in line with the *A. pullulans* descriptions by Barnett and Barry [22], Domsch et al. [23], and Hermanides-Nijhof [24].

### 3.2. Molecular Identification of A. pullulans

**Figure 2:**
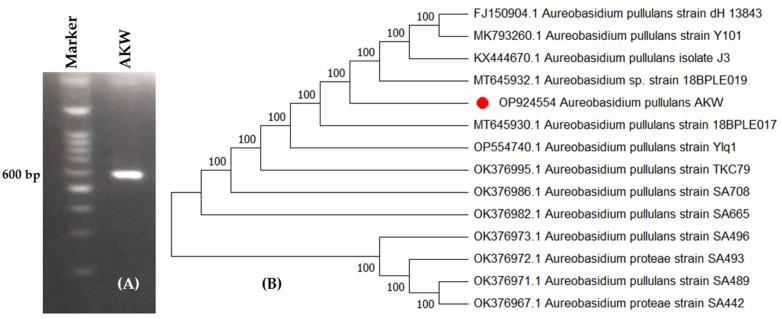
Agarose gel electrophoresis of the amplified bands of PCR product (**A**) and molecular phylogenetic tree based on partial ITS sequence (**B**) of *A. pullulans* AKW (OP924554), marked by a red dot.

*A. pullulans* AKW was molecularly identified based on ITS. The method compares the sequence of the 18S rRNA gene after it has been amplified by PCR. From BLAST results, the endophytic *A. pullulans* AKW displayed high similarity with the previously similar strains on the GenBank. The phylogenetic tree (Figure 2) shows the fungus as *A. pullulans* AKW, which was in agreement with the morphological identification. The fungal strain was registered on GenBank with accession number OP924554. *A. pullulans* AKW belongs to Eukaryota; Fungi; Dikarya; Ascomycota; Pezizomycotina; Dothideomycetes; Dothideomycetidae; Dothideales; Saccotheciaceae; and *Aureobasidium*.

Molecular identification, due to its high accuracy, is commonly used to quickly identify filamentous fungi in different taxonomic categories. This technique involves amplifying and comparing the sequence of the 18s rRNA gene through the ITS region using PCR. The uniform fragment size of the ITS in many fungal clusters makes nucleotide sequencing of ITS essential for detecting interspecific and sometimes intraspecific variation [31].

The well-known sequence, used for constructing phylogenies, is accurately labeled and demonstrates a strong connection to similar fungal strains in the GenBank. The ITS sequence is consistent across a broad range of fungal groups, making it valuable in detecting interspecific and sometimes intraspecific differences among organisms [32]. Additionally, the sequences of the non-functional ITS region are frequently highly diverse among fungal species, making it sufficient for identifying fungi at the species level [31,33]. Technically, the rDNA’s repeated nature makes the ITS regions easy to amplify from a small DNA sample [31]. Thus, ITS’s nucleotide sequencing is considered a fast and highly accurate identification approach compared to other markers. This approach can also be used for barcoding a wide range of fungal species [34,35].

### 3.3. Pullulan Biosynthesis Using Taguchi Approach

The pullulan biosynthesis process by *A. pullulans* was scaled up utilizing the Taguchi archetype by testing seven continuous variables that influence the biosorption process at three levels each (3^7^). The Taguchi orthogonal array (L_27_) generated 27 trials. The actual levels of the seven tested factors, together with the orthogonal array of L_27_, are presented in Table 1.

The Taguchi method was utilized to find the best conditions to enhance the pullulan biosynthesis process. Its orthogonal array design allowed for the efficient development of optimal conditions using the lowest number of trials, thereby saving both time and effort [15,17]. The orthogonal arrays of the seven control factors and the corresponding pullulan biosynthesis laboratory data of the 27 trials are presented in Table 1. As it appeared, the experiment was repeated in three separate runs; then, the data were statistically analyzed to estimate the mean of pullulan biosynthesis and *S/N* ratio.

The calculated SD values were very small, indicating the accuracy of the repeated runs. Normally, Taguchi analysis often begins with the calculation of the *S/N* ratio to identify the control factor(s) that reduces variability, the *S/N* ratio was optimized based on the larger-is-better role, which is used to amplify the pullulan biosynthesis process, thus the level of control factor(s) that maximize pullulan biosynthesis and have no or small impact on the *S/N* ratio is determined. Accordingly, the highest pullulan biosynthesis and the *S/N* ratio (17.18) were achieved by trial L_7_ (7.23%).

#### 3.3.1. Determination of Significant Factors

Firstly, the Taguchi model was utilized to calculate the fitted (predicted) values of pullulan biosynthesis. The fitted values were so adjacent to those of the actual ones; thus, the errors (residuals) were low, evidencing the model’s accuracy. Secondly, the overall design aptness was tested by measuring the determination coefficient (R^2^) and adjusted R^2^, being 0.9937 and 0.9863, respectively. Both are used for the model assessment. The present R^2^ and adjusted R^2^ values are close to one, being, concluding the accuracy of the Taguchi model.

It is already known that both kinds of R^2^ range from 0 to 1, and the closer to 1, the accuracy of the modeling capability [36]. If their values are ≥0.9, the model is adequately significant; in any case, R^2^ should not be <0.75 [37,38]. The R^2^ value assesses how much the response of pullulan biosynthesis varies based on changes in the amount of the tested factors. Adding more factors leads to a constant increase in R^2^, but this doesn’t account for the significance of the factors. The adjusted R^2^ solves this problem by considering the number of factors in the model. Unlike R^2^, adjusted R^2^ adjusts sensibly when adding significant factors only to the model, making it a better indicator of the model’s goodness of fit.

The ANOVA was performed on data from the Taguchi matrix to determine which of the seven medium variables has a significant influence on pullulan biosynthesis by *A. pullulans* (Table 2). Initially, the null hypothesis assumes that all terms have an equal effect and there is no relationship between the tested variables and the pullulan biosynthesis process.

The probability value was considered as a diagnosis tool for rating the significance. If the *p*-value < 0.05, the variable is considered significant in the pullulan biosynthesis process. On this basis, the seven investigated control factors had a significant effect on pullulan biosynthesis, except yeast extract, which recorded a *p*-value equal to 0.793 [38].

#### 3.3.2. Assignment of the Optimum Factors’ Levels

**Figure 3:**
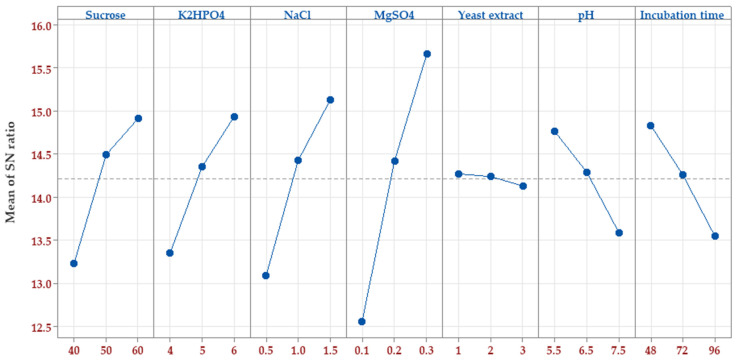
The plot of the *S/N* ratio effect based on larger-is-better for pullulan biosynthesis by *A. pullulans*.

Following the determination of significant factors, the next step was to assign the ideal value to each factor. To resolve this point, The *S/N* ratio was estimated for each factor level (Figure 3). The goal of optimizing process parameters was to improve the *S/N* ratio for better outcomes. The optimal level for each control factor that reduces noise variability and maximizes pullulan biosynthesis by *A. pullulans* was determined. The peak *S/N* ratio for each control factor was at the highest level of four factors, i.e., sucrose (60 g/L), K_2_HPO_4_ (6 g/L), NaCl (1.5 g/L), and MgSO_4_ (0.3 g/L), whereas at the lowest level of yeast extract (1 g/L), pH (5.5), and incubation time (48 h).

The Taguchi method is used to evaluate the mean output of each run in the inner set and determine the variability using the *S/N* ratio, which varies accordingly. The *S/N* ratio gauges the output’s variation in relation to the target value under varying noise conditions. Taguchi’s design is robust and can easily pinpoint the control factors (7 in this instance) that minimize variability in a process by reducing the impact of uncontrollable noise factors. Taguchi’s design philosophy involves inducing variability by manipulating noise factors during experimentation, making it easier to identify control factor settings that increase process resilience to noise factor variation. A higher *S/N* ratio signifies optimal control factor settings in reducing the impact of noise (non-controlled) factors [16,39,40].

The *S/N* ratio response of pullulan biosynthesis for each level of each control factor was calculated, then the delta (a measure of the range between the uppermost and lowermost average response values) for each factor was estimated. Accordingly, the rank was determined, and the highest delta value designates the relative importance of the factor on the *S/N* ratio (Table 3).

The results show that MgSO_4_ (delta 3.11, rank 1) has the major effect on the *S/N* ratio, followed by NaCl (delta 2.04, rank 2), sucrose (delta 1.69, rank 3), K_2_HPO_4_, (delta 1.59, rank 4), incubation time (delta 1.29, rank 5), initial pH (delta 1.17, rank 6), and finally yeast extract (delta 0.14, rank 7). The average mean response of the seven tested factors came in line with the *S/N* ratio, with an exception in the order or rank of both sucrose and K_2_HPO_4_. Such mathematical calculations concluded satisfactory accuracy of both the *S/N* ratio and mean analysis of the seven tested input variables.

Eventually, Taguchi orthogonal array was utilized to determine the optimal operating conditions of the parameters with the greatest impact on the target output. Further, the optimum level of each variable was also assigned. Before approving these results, the residual analysis is usually explored.

#### 3.3.3. Residual Analysis

**Figure 4:**
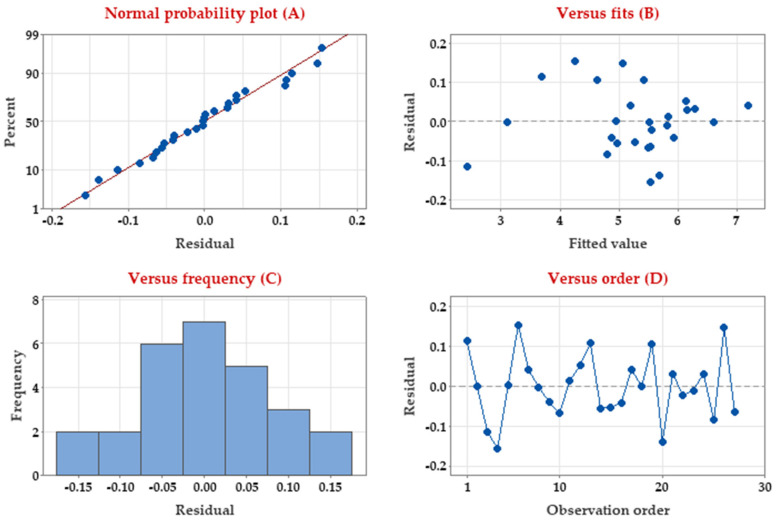
Residual analysis of pullulan production by *A. pullulans* based on the Taguchi model.

Residuals (errors) represent the discrepancy between the predicted output from a model and the actual measured output. Thus, residuals represent the portion of the validation data not explained by the model. Residuals were estimated to determine whether they are negatively affecting the Taguchi model and to verify whether the model meets the assumptions of the data analysis of pullulan biosynthesis. Four residual analyses were depicted (Figure 4).

The normality of residuals was assessed (Figure 4A) to verify the normal distribution of the residuals. As shown, the normal probability plot of the residuals follows a straight line without outlier points, confirming the non-normality. Residual vs. fit values (Figure 4B) show the random distribution of the residuals and further have constant variance i.e., distributed eventually on both sides of 0 with no identifiable arrangement. Another, depicting the histogram of residuals frequency (Figure 4C), revealed no skewness or outliers of the residuals. Finally, when residuals are displayed in time order, i.e., plotting the residuals vs. order of run (Figure 4D), the pattern verifies that the residuals are independent and randomly fall around the center line with no specific pattern or tendencies. All previous residual analyses supported the normality of the errors (residuals) and the appropriateness of the Taguchi design in modeling pullulan biosynthesis by *A. pullulans*.

Taguchi’s experimental design is a robust approach that forms the cornerstone of the Taguchi method, enabling accurate prediction of multi-variable processes with minimal experimental trials [16,39,40]. This work demonstrates the effectiveness of using the Taguchi method to identify the optimal combination of seven variables to maximize pullulan. Based on Taguchi data, the seven examined variables exhibited various degrees of significance and importance. Thus, the null hypothesis was disproven since the alternative hypothesis indicates the existence of significant variation caused by the parameters studied.

### 3.4. Decision Tree Learning Algorithm

The data from Taguchi experimental design (Table 1) were used for further investigation using the DT learning algorithm. The data were split 70/30% training/test sets. Upon performing the decision tree learning process, the fitted and residual values were estimated together with the terminal nodes.

The DT is a possible solution to a problem based on given conditions. It starts with a single box and ends up in numerous branches and roots, i.e., numerous solutions. It is a type of supervised learning algorithm that has target variables. To select solutions, DT creates classifications, which are applied to both categorical and continuous variables. Using a DT, the population or samples can be split into two or more homogeneous sets. These homogeneous sets are constructed based on the most significant differentiator on input variables [19,20].

Little or no information is available about the application of DT in the fermentation processes for the determination of important factors. However, herein DT was used to optimize the process parameters of the fermentation medium by analyzing the relationship between the process parameters and the pullulan yield. This was done by training the DT to predict which of the seven process parameters is optimal for fermentation conditions. The DT was trained using the obtained data collected from pullulan production. The decision tree was used to identify the most important process parameters and identify the optimal range of values for these parameters.

#### 3.4.1. Decision Tree Selection

**Figure 5:**
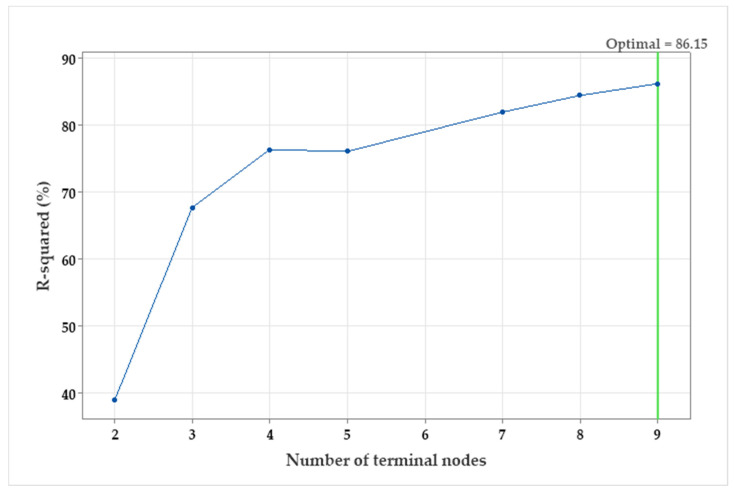
The R^2^ vis number of terminal nodes plot generated by a decision tree for pullulan production by *A. pullulans*.

The R^2^ vis number of the terminal node was plotted (Figure 5) to find out the smallest regression tree that maximizes the R^2^ value. The R^2^ values for nine trees and nine terminal nodes were generated from the validation samples. The R^2^ values of the validation samples typically level off (plateau) and eventually start to decline as the tree grows larger. The optimal regression tree that maximizes the R^2^ value is 91.51 (for training) and 86.15% (for testing). The tree is optimal as the selection criterion was to create the smallest tree with an R^2^ value within a standard deviation of 1 of the maximum R^2^ value.

Because the chart shows that the R^2^ values are relatively increased (more than a standard deviation of 1) up to the end of the 9th node; therefore, there is no need to look at the performance of some of the smaller trees that are like the optimal tree. Typically, a tree with fewer terminal nodes (such as the current nine-terminal node tree) provides a clearer understanding of how each predictor variable influences the response values. Additionally, a smaller tree simplifies identifying a few key predictors for additional research.

**Figure 6:**
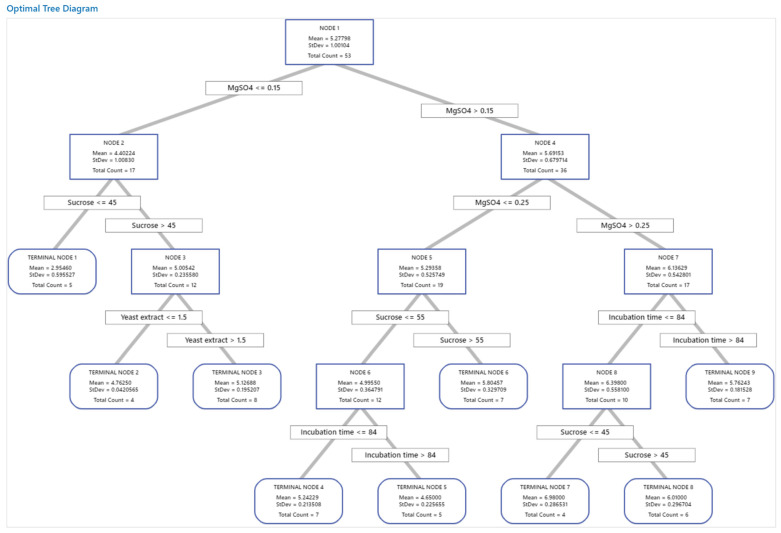
The decision tree with a depth of four of the pullulan biosynthesis using *A. pullulans*. A total of 81 data sets from Taguchi’s experimental design were employed in the decision tree learning algorithm to predict pullulan yield. The data used for node selection are introduced in the squares. The squares involved in the paths of the best variable mixtures for pullulan biosynthesis were predicted with the decision tree.

The tree was constructed and investigated for the distinction of terminal nodes on the diagram (Figure 6). The tree diagram shows all 81 cases from the full data set. The target was to find the largest mean with the lowest standard deviations.

The first node used 53 cases and is split at two levels of MgSO_4_, i.e., ≤0.15 and >0.15 g/L. Node 2 has 17 cases where the mean for the node (4.40 g/L pullulan) is less than the overall mean (5.28 g/L pullulan), with a standard deviation of 1.01, which is >the overall standard deviation. Node 4 has 36 cases with a low standard deviation, 0.68, which is < the overall standard deviation because a split yields a purer node. The mean (5.70 g/L pullulan) for the node is more than the overall mean. This node split MgSO_4_ into two levels at <0.25 and >0.25 g/L. Node 7 used 17 cases and showed further improvement in the mean and standard deviation. This node was split based on incubation time into ≤84 and >84 h. Node 8 used 10 cases and also showed improvement and split into two terminal nodes (7 and 8) based on sucrose concentration. Terminal node 7 has the highest mean, with only four cases. Accordingly, terminal node 7 showed the highest mean value of pullulan (6.98 ± 0.29 g/L). The node rules were sucrose ≤ 45, MgSO_4_ > 0.25, and incubation time ≤ 84; these rules were achieved in run no. L_7_, which also acts as a terminal node in the decision tree. On the other side, terminal node 1 has the smallest mean (2.95 g/L), suggesting that the data in terminal node 1 are probably skewed.

The error statistics of the decision tree learning model were calculated. The accuracy of the resulting tree was indicated with low MSE, being 0.0850 and 0.1397, and RMSE being 0.2916 and 0.37380.3738, for training and testing processes, respectively.

#### 3.4.2. The Relative Variable Importance

**Figure 7:**
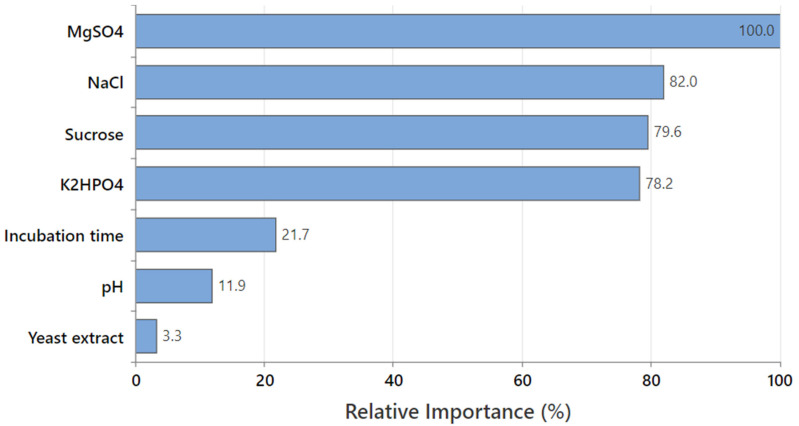
The relative importance of the seven tested factors on pullulan production based on the decision tree learning algorithm.

The relative variable importance is quantified as the increasing percentage relative to the leading predictor (variable). The leading predictor has a relative importance of 100%, thus, the other variables are standardized relative to the leading predictor; accordingly, the importance of each variable could be easily interpreted. The chart (Figure 7) of the relative variable importance was used to detect which predictor is the most important to the tree. The most important (leading) variable is determined as the one with the highest score of improvement (and thus considered 100% important), and the rest of the variables are ranked accordingly. Variables that are considered important are primary or surrogate splitters in a decision tree. The most vital variable always has 100% relative importance (MgSO_4_ in the current study). The non-important variable does not appear in the tree.

As MgSO_4_ was the most important predictor variable with a 100% contribution in pullulan biosynthesis, the other variables were compared to MgSO_4_ to determine their relative importance. Consequently, NaCl (82.0%), sucrose (79.6%), and K_2_HPO_4_ (78.2%) showed higher relative importance than incubation time (21.7%) and pH (11.9%); the yeast extract was the lowest variable.

Although the positive importance of the seven variables, the relative ranking informs about the number of variables to regulate or keep track of in pullulan production. Significant decreases in the relative importance values between variables can assist in determining which variables should be regulated or monitored. In this connection, four variables have the highest relative important values that are relatively close together before a drop to 21.7% in relative importance for the incubation time. It is worth mentioning that the relative variable importance is in harmony with that obtained by Taguchi’s experimental data, confirming the accuracy of the experimental design and the obtained data.

### 3.5. Validation of Taguchi and DT Models

The prediction level of each of the seven tested factors was determined by both models (Table 4). The laboratory confirmation experiments were conducted in triplicate to verify the optimal process conditions calculated from both design models. The obtained experimental data confirmed and verified the fitness of both models when compared with predicted values.

The maximum pullulan biosynthesis by *A. pullulans* at the speculative values of the optimal levels of the seven tested variables was 7.17%, with a *S/N* ratio of 17.64. Under laboratory experimental conditions, these estimated values were evaluated. The experimental value of pullulan biosynthesis was obtained at 7.23 ± 0.13%. Similarly, to check the forecast capacity of the DT model, the actual value was calculated to be 7.11 ± 0.07 compared with the predicted one. This value was separated at the seventh node of the DT.

The actual values showed accuracy and were very close and obey to the predicted value by both models. However, from the economic point of view, the performance of the DT model was better than the Taguchi model since it takes only 40 g/L of sucrose compared to 60 g/L in the case of Taguchi to yield a similar yield of pullulan.

The relative importance and optimum level for each of the investigated variables varied accordingly. However, each variable plays a distinct role in pullulan biosynthesis. The most important one is magnesium sulfate, which is essential for the growth and survival of fungi. It acts as a source of magnesium involved in many metabolic processes, including energy production and DNA synthesis. Its sulfur moiety provides sulfur, which is an important component of amino acids and coenzymes [41,42]. Magnesium sulfate is commonly used as a co-factor in pullulan production. It acts as a key ionic activator in the formation of pullulan polysaccharides by the enzyme pullulanase. Additionally, magnesium sulfate can help regulate the osmotic pressure and pH in the reaction mixture, leading to improved pullulan yield and quality [43].

Sodium chloride came next in terms of relative importance. The rates of both specific glucose consumption and, consequently, the specific pullulan production rate improved due to the presence of NaCl, causing greater pullulan yield. Moreover, NaCl stimulates glucosyltransferase and α-phosphoglucose mutase (involved in pullulan biosynthesis) and also stimulates α-amylase (the degraded pullulan), upregulates the transcriptional levels of *fks*, *pgm1*, and *amy2* genes, enhances the driving force for ATP supply, and helps to maintain intracellular uridine diphosphate-glucose at a high level in the fungal cells [44,45]. However, NaCl is essential for fungal growth, as it helps regulate the osmotic pressure of the growth medium. Fungi require an appropriate balance of solutes, including sodium and chloride ions, to maintain cellular turgor and facilitate the proper uptake of nutrients. Sodium ions play a role in regulating fungal osmotic pressure, while chloride ions are involved in metabolic processes and in maintaining the stability of cell membranes. However, too much NaCl can be toxic to fungi and inhibit their growth, so it is important to regulate its concentration in the growth medium [46,47].

Sucrose was the third important factor. An earlier study reported that the minimum response of pullulan production happened at the lowest level of sucrose; as the sucrose concentration increased, a considerable increment in pullulan biosynthesis occurred, indicating the significant influence of sucrose concentration on pullulan production. However, a higher concentration of sucrose led to a decrease in pullulan production due to low water activity and the osmotic impact caused by elevated sucrose levels [28].

Dipotassium hydrogen phosphate is used as a food additive and has been approved as generally recognized as safe. Besides acting as a crucial nutrient of potassium and phosphorus source for fungal growth, cell division, and metabolic processes, it also acts as a pH buffer to regulate and stabilize the medium pH, helping to maintain a stable pH environment for optimal fungal growth [48].

Incubation time is a determinant and key factor in pullulan production, as it affects the growth and metabolic activity of the microorganisms and the synthesis of pullulan. Obviously, our endophytic *A. pullulans* strain showed a relatively short incubation period (48 h) compared with the previously stated time [28,49]. In addition, the optimal incubation time varies depending on the microorganism and the conditions of the reaction, but generally, longer incubation times lead to increased pullulan yields. However, excessively long incubation times can result in reduced pullulan yields due to substrate depletion and the accumulation of inhibitory substances. It is, therefore, important to carefully control the incubation time to obtain optimal pullulan yields [45].

The degree of pH plays a crucial role in pullulan production, affecting both the growth of the microorganisms and the production of pullulan. Optimal pH conditions can vary depending on the specific microorganisms used, but generally, a slightly acidic to neutral pH (6.0–7.0) is favored. This range of pH values provides the microorganisms with the necessary conditions to carry out their metabolic activities, leading to the synthesis and accumulation of pullulan. Additionally, controlling pH can also help regulate other factors, such as osmotic pressure and the availability of essential ions for the production of pullulan [45].

Although yeast extract was ranked last in terms of relative importance, it is a common ingredient in the production of pullulan, serving as a source of essential nutrients (amino acids, vitamins, and trace elements) for the pullulan-producing microorganisms, thus plays an important role in promoting the growth and metabolic activity of the fungus, ultimately leading to the synthesis and accumulation of pullulan [45].

### 3.6. The Structure Characterization of Pullulan

The structure of the purified pullulan isolated from *A. pullulans* was interpreted in this work through the spectroscopic analysis involving the usage of two distinctive spectroscopic performances (FT-IR and ^1^H-NMR spectra).

#### 3.6.1. The FT-IR Spectroscopy

FT-IR spectroscopy is a type of infrared spectroscopy that uses Fourier-transform techniques to analyze the infrared light absorbed or emitted by a sample. It is used to identify chemical compounds and analyze the molecular structure of materials.

**Figure 8:**
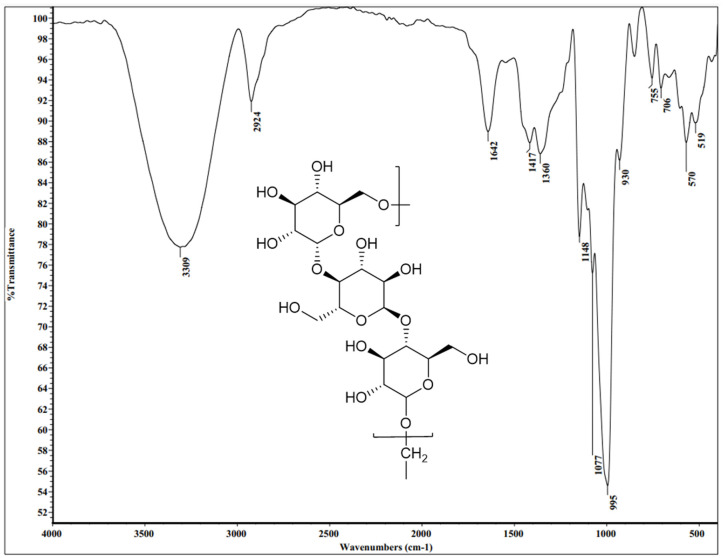
The FT-IR chart of *A. pullulans* AKW pullulan with a frequency range from 4000 to 350 cm^−1^.

The FT-IR spectral analysis was implemented to investigate the structural elucidation of the produced pullulan skeleton. Generally, the spectral data that was recorded (Figure 8 and Table 5) well-established the pullulan structure. As shown, the FT-IR spectral data (KBr, *ν* cm^−1^) revealed a sharp absorption band at 3309 cm^−1^ attributed to the stretching vibration of bonded and non-bonded OH groups, which are ascribed to the alcoholic or aliphatic hydroxyl chains. The absorption band that was recorded at 2924 cm^−1^ is attributed to medium C-H stretching owing to the presence of the *sp*^3^ C-H bond. Additionally, the absorption band situated at 1642 cm^−1^ is ascribed to the weak stretching vibration of O-C-O groups, which verified the presence of aliphatic ether moiety in the structural skeleton. Nevertheless, this result is well-matched with that reported study, which specified the value of the characteristic stretching vibration of O-C-O groups at 1636 cm^−1^ [50]. The absorption band that appeared at 1417 cm^−1^ is attributed to the medium C-H bending of the alkane chain of the *sp*^3^ C-H bond. Also, a medium absorption band due to the C-O-H group appeared at 1360 cm^−1^, signifying the presence of aliphatic ether moiety. Other types of pullulan were correspondingly perceived in the spectra comprising the C-O-C stretching absorption band at 1148 cm^−1^ and the C-O stretching absorption bands at 1077 and 995 cm^−1^. The strong absorption band due to the C-O-C group that appeared at 1148 cm^−1^ is typically well-matched with several previous data of FT-IR spectra [50,51,52]. In particular, a weak band that appeared in the FT-IR chart at 850 cm^−1^ supported the α-configuration of *α*-D-glucopyranose fragments in the pullulan skeleton. Additionally, the basic linkage of glucopyranose units to construct the pullulan framework appeared in the recorded data at 706, 755, and 930 cm^−1^—these values estimated the linkage of the units was accomplished through *α*-(1,4) and *α*-(1,6)-D-glycosidic bonds [53].

#### 3.6.2. The ^1^H-NMR Spectroscopy

The ^1^H-NMR is a type of nuclear magnetic resonance spectroscopy that specifically measures the magnetic properties of hydrogen nuclei (protons) in a sample. It is used to determine the molecular structure of organic compounds and to study the properties of molecules in solution. The ^1^H-NMR spectrum provides information about the number of hydrogen atoms, their chemical environment, and their relative orientation in the molecule.

**Figure 9:**
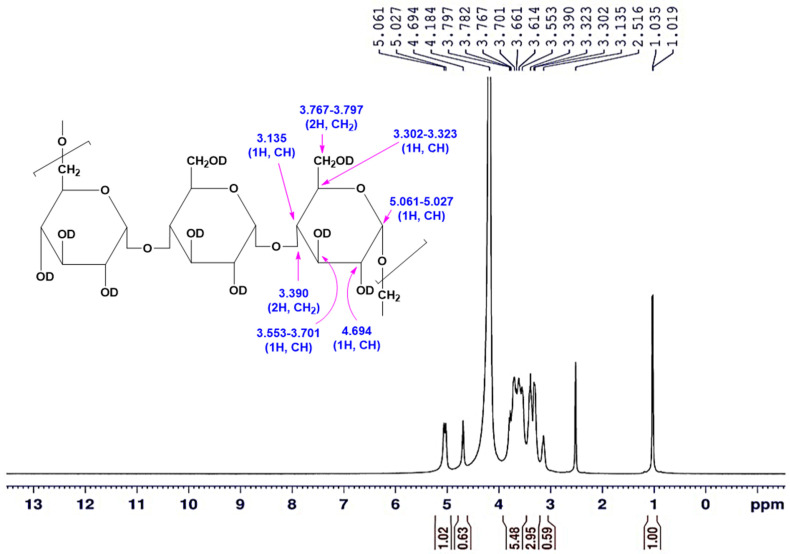
The ^1^H-NMR (DMSO-*d*6/D_2_O) spectrum of the *A. pullulans* AKW pullulan.

**Figure 10:**
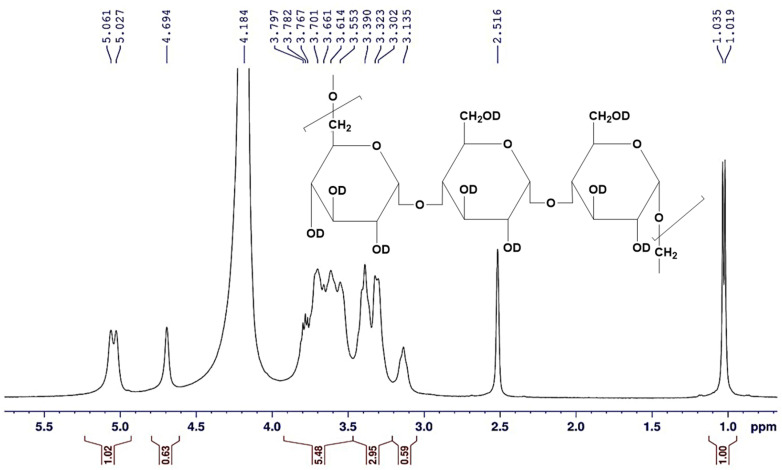
The magnification of the aliphatic region in the ^1^H-NMR (DMSO-*d*6/D_2_O) spectrum of the *A. pullulans* AKW pullulan.

The ^1^H-NMR spectrum was performed for pullulan to characterize the structural framework. The protons of hydroxyl groups are exchangeable with D_2_O owing to dissolving the pullulan sample in a mixture of DMSO-*d*_6_/D_2_O to improve the solubility. A one-dimensional ^1^H-NMR spectrum was applied for the tested sample, which verified signals for the total nine protons for each glucose unit, excluding the exchangeable hydroxyl protons with D_2_O. The data of the ^1^H-NMR spectrum (Figure 9 and Figure 10) demonstrated that the signal attributed to the CH proton at the C_4_ position appeared at *δ* = 3.135 ppm. The recorded signals appeared in the downfield region (aliphatic region) between *δ* = 3.135 to 5.027 ppm deducing the protons on carbon atoms adjacent to high electronegative atoms verified the distinctive chemical location of a carbohydrate core. Consequently, the total integrated protons from the ^1^H-NMR chart indicated the presence of carbohydrate polymer chains with three units of the sugar moiety. The signals at *δ* = 5.027–5.061 ppm supported the α-1,6 and α-1,4 glycosidic bonds at the C_1_ position of the sugar moiety, as well as the formation of (1→4) and (1→6) linkages. The data verified the presence of two singlet signals at *δ* = 3.135 and 4.694 ppm attributed to the protons attached to the C_4_, and C_2_ positions, respectively. Correspondingly, the CH and CH_2_ protons appeared at 3.302–3.323, 3.553–3.701, and 3.767–3.797 ppm as a doublet or doublet of doublet signals are attributed to the protons attached to the C_3_, C_5_, and C_6_ carbons. The methine proton at the C_1_ position appeared more de-shielded at 5.061–5.027 ppm owing to the impact of the two adjacent oxygen atoms (high electronegative atom) to the carbon atom at the C_1_ position.

In comparison to the studies [53,54,55] that previously reported on the chemical structure elucidation of pullulan by ^1^H-NMR spectroscopy, typical data were recorded herein, excluding the signals due to the hydroxyl groups exchanged with D_2_O. In addition, the comparison of our data with commercial pullulan spectra [29,49] confirmed the structure of the current pullulan.

Several investigations have been conducted on pullulan production by *A. pullulans*. Overall, the results of previous studies have varied depending on the conditions used. However, these studies have provided valuable insights into the optimization of pullulan production by *A. pullulans* and the potential use of alternative nutritional and fermentation conditions for pullulan production [56]. One earlier study reported that the optimal conditions for pullulan biosynthesis by *A. pullulans* were at 28 °C and an agitation rate of 200 rpm. Under these conditions, the yield of pullulan was 40.1 g/L [12]. In addition, the statistical optimization of the central composite design was applied for using molasses as a carbon source for pullulan production by *A. pullulans*, with a yield of 45 g/L [13]. A more recent study investigated the use of soybean meal hydrolysate as a nitrogen source and found that pullulan production by *A. pullulans* reached 59.8 g/L [14].

Our study, on the other hand, represents the state-of-the-art for the biosynthesis of pullulan by the endophytic strain that is capable of producing 72.3 g/L, representing about a 1.5-fold increase over most of the previous investigation. This may also be due to the novel application of Taguchi’s design and the DT learning algorithm.

## 4. Conclusions

In sum, our results provide a concise and insightful description of applying both the Taguchi and DT learning algorithm paradigms in the biosynthesis process of pullulan by the new endophytic candidate, *A. pullulans* AKW. Another important conclusion that can be drawn is the relatively short fermentation period compared with previous studies on a more simplified medium. However, further research is encouraged for additional studies on using artificial intelligence to optimize the fermentation conditions for the maximization of pullulan biosynthesis. Other studies are also encouraged to focus on its biosynthesis regulation under the current fermentation circumstances.

## Figures and Tables

**Table 1:** Orthogonal array and predicted values of Taguchi’s L_27_ (3^7^) for optimization of pullulan biosynthesis by *A. pullulans*, as well as the predicted and residual values and terminal nodes of the decision tree learning algorithm at each data point. The experiment was repeated in three separate runs denoted by different colors.

Run	Tested Variable							Pullulan Biosynthesis (%)						
	Nutritional Component (g/L)					pH	Incubation (h)	Taguchi			Decision Tree			
	Sucrose	K_2_HPO_4_	NaCl	MgSO_4_	Yeast Extract			Mean	*S/N* Ratio	Fitted	Type	Fitted	Residual	Terminal Node
L1	40	4	0.5	0.1	1	5.5	48	3.81 ± 0.23	11.59	3.70	Test	2.95	1.04	1
L2	40	4	0.5	0.1	2	6.5	72	3.12 ± 0.10	9.86	3.12	Test	2.95	0.16	1
L3	40	4	0.5	0.1	3	7.5	96	2.32 ± 0.01	7.30	2.43	Training	2.95	−0.62	1
L4	40	5	1.0	0.2	1	5.5	48	5.37 ± 0.07	14.60	5.53	Test	5.24	0.19	4
L5	40	5	1.0	0.2	2	6.5	72	4.94 ± 0.05	13.88	4.94	Training	5.24	−0.25	4
L6	40	5	1.0	0.2	3	7.5	96	4.41 ± 0.10	12.88	4.26	Test	4.65	−0.19	5
L7	40	6	1.5	0.3	1	5.5	48	7.23 ± 0.09	17.18	7.19	Training	6.98	0.35	7
L8	40	6	1.5	0.3	2	6.5	72	6.60 ± 0.17	16.39	6.61	Test	6.98	−0.57	7
L9	40	6	1.5	0.3	3	7.5	96	5.88 ± 0.09	15.39	5.92	Training	5.76	0.15	9
L10	50	4	1.0	0.3	1	6.5	96	5.43 ± 0.11	14.69	5.50	Training	5.76	−0.26	9
L11	50	4	1.0	0.3	2	7.5	48	5.84 ± 0.08	15.33	5.83	Training	6.01	−0.26	8
L12	50	4	1.0	0.3	3	5.5	72	6.19 ± 0.05	15.84	6.14	Test	6.01	0.13	8
L13	50	5	1.5	0.1	1	6.5	96	4.74 ± 0.06	13.51	4.63	Test	4.76	−0.09	2
L14	50	5	1.5	0.1	2	7.5	48	4.91 ± 0.07	13.81	4.96	Training	5.13	−0.21	3
L15	50	5	1.5	0.1	3	5.5	72	5.22 ± 0.04	14.35	5.27	Training	5.13	0.12	3
L16	50	6	0.5	0.2	1	6.5	96	4.83 ± 0.06	13.67	4.87	Training	4.65	0.23	5
L17	50	6	0.5	0.2	2	7.5	48	5.24 ± 0.05	14.39	5.20	Training	5.24	0.04	4
L18	50	6	0.5	0.2	3	5.5	72	5.51 ± 0.09	14.82	5.51	Training	5.24	0.19	4
L19	60	4	1.5	0.2	1	7.5	72	5.52 ± 0.05	14.84	5.42	Training	5.80	−0.31	6
L20	60	4	1.5	0.2	2	5.5	96	5.54 ± 0.06	14.86	5.67	Test	5.80	−0.32	6
L21	60	4	1.5	0.2	3	6.5	48	6.18 ± 0.12	15.81	6.15	Training	5.80	0.32	6
L22	60	5	0.5	0.3	1	7.5	72	5.53 ± 0.14	14.85	5.56	Test	6.01	−0.63	8
L23	60	5	0.5	0.3	2	5.5	96	5.80 ± 0.12	15.27	5.81	Test	5.76	−0.06	9
L24	60	5	0.5	0.3	3	6.5	48	6.32 ± 0.10	16.01	6.28	Test	6.01	0.29	8
L25	60	6	1.0	0.1	1	7.5	72	4.72 ± 0.08	13.48	4.81	Training	4.76	−0.06	2
L26	60	6	1.0	0.1	2	5.5	96	5.21 ± 0.10	14.34	5.07	Test	5.13	0.07	3
L27	60	6	1.0	0.1	3	6.5	48	5.48 ± 0.15	14.76	5.54	Training	5.13	0.31	3
L1	40	4	0.5	0.1	1	5.5	48				Training	2.95	0.95	1
L2	40	4	0.5	0.1	2	6.5	72				Training	2.95	0.27	1
L3	40	4	0.5	0.1	3	7.5	96				Training	2.95	−0.64	1
L4	40	5	1.0	0.2	1	5.5	48				Test	5.24	0.14	4
L5	40	5	1.0	0.2	2	6.5	72				Test	5.24	−0.35	4
L6	40	5	1.0	0.2	3	7.5	96				Training	4.65	−0.18	5
L7	40	6	1.5	0.3	1	5.5	48				Test	6.98	0.19	7
L8	40	6	1.5	0.3	2	6.5	72				Training	6.98	−0.33	7
L9	40	6	1.5	0.3	3	7.5	96				Training	5.76	0.02	9
L10	50	4	1.0	0.3	1	6.5	96				Training	5.76	−0.28	9
L11	50	4	1.0	0.3	2	7.5	48				Test	6.01	−0.12	8
L12	50	4	1.0	0.3	3	5.5	72				Training	6.01	0.19	8
L13	50	5	1.5	0.1	1	6.5	96				Training	4.76	−0.01	2
L14	50	5	1.5	0.1	2	7.5	48				Training	5.13	−0.30	3
L15	50	5	1.5	0.1	3	5.5	72				Test	5.13	0.11	3
L16	50	6	0.5	0.2	1	6.5	96				Training	4.65	0.18	5
L17	50	6	0.5	0.2	2	7.5	48				Training	5.24	−0.05	4
L18	50	6	0.5	0.2	3	5.5	72				Test	5.24	0.26	4
L19	60	4	1.5	0.2	1	7.5	72				Training	5.80	−0.30	6
L20	60	4	1.5	0.2	2	5.5	96				Training	5.80	−0.27	6
L21	60	4	1.5	0.2	3	6.5	48				Training	5.80	0.30	6
L22	60	5	0.5	0.3	1	7.5	72				Training	6.01	−0.44	8
L23	60	5	0.5	0.3	2	5.5	96				Training	5.76	0.17	9
L24	60	5	0.5	0.3	3	6.5	48				Training	6.01	0.22	8
L25	60	6	1.0	0.1	1	7.5	72				Training	4.76	0.05	2
L26	60	6	1.0	0.1	2	5.5	96				Training	5.13	0.19	3
L27	60	6	1.0	0.1	3	6.5	48				Test	5.13	0.51	3
L1	40	4	0.5	0.1	1	5.5	48				Test	2.95	0.60	1
L2	40	4	0.5	0.1	2	6.5	72				Training	2.95	0.06	1
L3	40	4	0.5	0.1	3	7.5	96				Test	2.95	−0.64	1
L4	40	5	1.0	0.2	1	5.5	48				Test	5.24	0.06	4
L5	40	5	1.0	0.2	2	6.5	72				Training	5.24	−0.30	4
L6	40	5	1.0	0.2	3	7.5	96				Training	4.65	−0.35	5
L7	40	6	1.5	0.3	1	5.5	48				Training	6.98	0.21	7
L8	40	6	1.5	0.3	2	6.5	72				Training	6.98	−0.23	7
L9	40	6	1.5	0.3	3	7.5	96				Training	5.76	0.19	9
L10	50	4	1.0	0.3	1	6.5	96				Test	5.76	−0.46	9
L11	50	4	1.0	0.3	2	7.5	48				Training	6.01	−0.12	8
L12	50	4	1.0	0.3	3	5.5	72				Test	6.01	0.23	8
L13	50	5	1.5	0.1	1	6.5	96				Training	4.76	0.03	2
L14	50	5	1.5	0.1	2	7.5	48				Training	5.13	−0.16	3
L15	50	5	1.5	0.1	3	5.5	72				Training	5.13	0.04	3
L16	50	6	0.5	0.2	1	6.5	96				Training	4.65	0.12	5
L17	50	6	0.5	0.2	2	7.5	48				Training	5.24	0.02	4
L18	50	6	0.5	0.2	3	5.5	72				Training	5.24	0.36	4
L19	60	4	1.5	0.2	1	7.5	72				Training	5.80	−0.22	6
L20	60	4	1.5	0.2	2	5.5	96				Test	5.80	−0.20	6
L21	60	4	1.5	0.2	3	6.5	48				Training	5.80	0.51	6
L22	60	5	0.5	0.3	1	7.5	72				Test	6.01	−0.36	8
L23	60	5	0.5	0.3	2	5.5	96				Training	5.76	0.02	9
L24	60	5	0.5	0.3	3	6.5	48				Training	6.01	0.41	8
L25	60	6	1.0	0.1	1	7.5	72				Test	4.76	−0.10	2
L26	60	6	1.0	0.1	2	5.5	96				Training	5.13	−0.01	3
L27	60	6	1.0	0.1	3	6.5	48				Test	5.13	0.22	3

**Table 2:** ANOVA of means of pullulan biosynthesis by *A. pullulans* as affected by the seven investigated factors.

Source	Freedom Degree	Sum of Square	Mean Square	F-Value	*p*-Value
Sucrose	2	2.50	1.25	87.62	0.000
K_2_HPO_4_	2	2.53	1.27	88.94	0.000
NaCl	2	4.86	2.43	170.44	0.000
MgSO_4_	2	13.04	6.52	457.60	0.000
Yeast extract	2	0.01	0.00	0.24	0.793
pH	2	1.70	0.85	59.83	0.000
Incubation time	2	2.15	1.08	75.47	0.000
Residual Error	12	0.17	0.01		
Total	26	26.96			
The determination coefficient (R^2^)		0.9937			
Adjusted-R^2^		0.9863			

**Table 3:** Response analysis of *S/N* ratio and means of Taguchi array of pullulan biosynthesis by *A. pullulans*.

*S/N* Ratio Response Analysis							
Level	Sucrose	K_2_HPO_4_	NaCl	MgSO_4_	Yeast Extract	Initial pH	Incubation Time
1	13.23	13.35	13.09	12.56	14.27	14.76	14.83
2	14.49	14.35	14.42	14.42	14.24	14.29	14.26
3	14.92	14.94	15.13	15.66	14.13	13.59	13.55
Delta	1.69	1.59	2.04	3.11	0.14	1.17	1.29
Rank	3	4	2	1	7	6	5
**Mean** **Response Analysis**							
1	4.854	4.883	4.720	4.391	5.243	5.543	5.597
2	5.324	5.249	5.289	5.282	5.245	5.292	5.263
3	5.589	5.634	5.757	6.093	5.278	4.931	4.906
Delta	0.736	0.750	1.037	1.702	0.035	0.612	0.691
Rank	4	3	2	1	7	6	5

**Table 4:** The predicted fermentation conditions were estimated based on the Taguchi and DT models as well as the predicted and actual values of pullulan biosynthesis by *A. pullulans*.

Model	Nutritional Component (g/L)					pH	Incubation (h)	Pullulan, %		S/N Ratio	Terminal Node
	Sucrose	K_2_HPO_4_	NaCl	MgSO_4_	Yeast Extract			Predicted	Actual		
Taguchi	60	4	1.5	0.3	1	5.5	48	7.17	7.23 ± 0.13	17.64	-
Decision tree	40	4	1.5	0.3	1	5.5	48	6.98	7.11 ± 0.07	-	7

**Table 5:** The FT-IR spectroscopic data of *A. pullulans* AKW pullulan.

Absorption (cm^−1^)	Appearance	Functional Group	Compound Class	Relative Intensity
3309	sharp	O-H stretching	Alcohol	0.250
2924	medium	C-H stretching	Alkane	0.066
1642	weak	O-C-O stretching	Aliphatic ether	0.073
1417	medium	C-H bending	Alkane	0.001
1360	medium	C-O-H	Aliphatic ether	0.097
1148	strong	C-O-C stretching	Aliphatic ether	0.047
1077	strong	C-O stretching	Alcohol	0.047
995	strong	C-O stretching	primary alcohol	0.034
930	strong	C-H bending	trisubstituted	0.034
755	strong	C-H bending	disubstituted	0.012
706	strong	C-H bending	disubstituted	0.012

## Data Availability

All data were reported in the paper.
